# Influence of the Bismuth Content on the Optical Properties and Photoluminescence Decay Time in GaSbBi Films

**DOI:** 10.1021/acsomega.3c05046

**Published:** 2023-09-18

**Authors:** Tristan Smołka, Michał Rygała, Joonas Hilska, Janne Puustinen, Eero Koivusalo, Mircea Guina, Marcin Motyka

**Affiliations:** † Laboratory for Optical Spectroscopy of Nanostructures, Department of Experimental Physics, Faculty of Fundamental Problems of Technology, Wrocław University of Science and Technology, Wybrzeże Wyspiańskiego 27, 50-370 Wrocław, Poland; ‡ Optoelectronics Research Centre, Physics Unit, Tampere University, Korkeakoulunkatu 3, 33720 Tampere, Finland

## Abstract

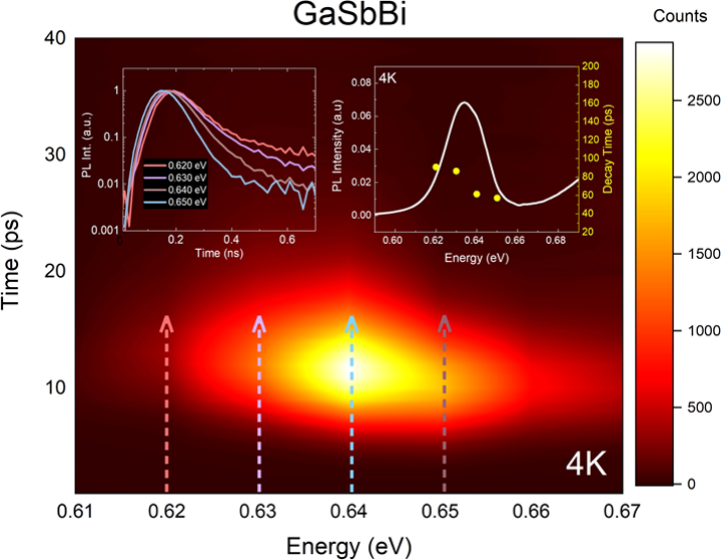

We report the optical properties of GaSbBi layers grown on GaSb (100) substrates with different bismuth contents of 5.8 and 8.0% Bi. Fourier-transform photoluminescence spectra were determined to identify the band gaps of the studied materials. Further temperature- and power-dependent photoluminescence measurements indicated the presence of localized states connected to bismuth clustering. Finally, time-resolved photoluminescence measurements based on single-photon counting allowed the determination of characteristic photoluminescence decay time constants. Because of the increasing bismuth content and clustering effects, an increase in the time constant was observed.

## 1 Introduction

Optical sensing systems for applications in the infrared spectrum typically consist of a laser source, operating preferably in single mode, such as diode lasers (DLs),^1^ quantum cascade lasers (QCLs),^2^ or interband cascade lasers (ICLs),^3^ and a detector, ideally working at room temperature or cooled thermoelectrically (TE). Another approach for detecting multiple gases simultaneously uses dual-comb spectroscopy, as it was conducted in laboratory conditions in ref.^4^ The generation of optical frequency combs can be achieved through the mode-locking technique.^5^ The fabrication of mid-infrared mode-locked lasers was only recently proven possible,^6^ and extensive work is still required for the development and engineering of new material systems.

In recent years, dilute bismide III–V compound semiconductors have attracted a lot of attention due to their unique properties of bandgap reduction and spin–orbit splitting energy increase.^7,8^ Furthermore, in comparison to the dilute III–V nitrides, they offer interesting benefits such as preserved electron mobility.^9^ Such properties make III–V Bi semiconductors attractive for optoelectronic devices operating in the near- and mid-infrared spectral ranges. However, the large size of the Bi atom and its nature to bond weakly to group III atoms makes it difficult to incorporate Bi into the III–V lattice, and thus Bi tends to segregate^10^ to the crystal growth surface and/or evaporate.^11^ A yet emerging alloy in the III–V Bi family is GaSbBi, which has attracted attention due to its potential applications in the mid-IR region. However, so far, most studies have focused on the growth and structural properties of the novel alloy, with only a few groups demonstrating in detail their optical properties. Recently, some attention has also been paid to the investigation of the carrier dynamics in GaInSbBi/GaSb quantum wells.^12^ In this study, we focus on the investigation of the photoluminescence (PL) lifetime in bulk crystal GaSbBi layers. The results of this work may prove valuable for further development of mid-infrared mode-lock lasers, where the cavity round-trip parameter is crucial for its operation. As it was shown in ref (13), passive mode locking is difficult to achieve in quantum cascade lasers (QCLs) due to its operation principle based on inter-subband transitions with a short upper-state lifetime (around 1 ps). Another important application where carrier dynamics are important is for fabricating fast semiconductor saturable absorber mirrors.^14^ These applications require certain parameters for the active region materials, which need to match values for creating mode-locked devices. Our studies focus on measuring materials in which interband transitions based on both electrons and holes could achieve certain PL lifetime values that might be suitable for achieving passive mode-locking.

## 2 Samples

In this paper, we present the optical properties of GaSbBi layers grown by molecular beam epitaxy (MBE). Two samples with different bismuth atom concentrations, sample A—5.8% and sample B—8.0%, were investigated. Both samples were grown on 500 μm-thick (100) n-GaSb substrates using the same growth procedure as in our earlier work.^11^ In short, the growth is initiated by depositing a surface-smoothing 100 nm-thick GaSb layer at ∼500 °C, followed by a temperature ramp-down sequence to ∼300 °C, with both temperatures estimated via pyrometry. The low temperature is required for Bi incorporation into the subsequently grown 150 nm-thick GaSbBi layer together with a low Sb-flux overpressure slightly above the stoichiometric 1:1 flux ratio. More details on the influence of growth conditions on Bi incorporation and the resulting structural properties of GaSbBi are presented elsewhere.^11^

## 3 Experimental Setup Description

To measure the Fourier-transform photoluminescence (FTPL) spectra, a vacuum-sealed Bruker Vertex 80v spectrometer was used. It operated in step-scan mode, with an external measurement chamber and an additional modulated pumping laser beam.^15,16^ The signal was gathered by a liquid-nitrogen-cooled InSb photodiode detector, while phase-sensitive detection of the optical response was performed using a lock-in amplifier. The excitation beam was provided by a 660 nm line of a laser diode modulated at a frequency of 800 Hz with a chopper.

Time-resolved photoluminescence measurements were recorded by a superconducting single photon detector (SSPD) with an NbN nanowire array (cutoff wavelength ∼2.3 μm) and TRIAX grating monochromator. The excitation beam (830 nm) was provided by a mode-locked Ti-Sapphire laser. The laser system generates trains of ∼140 fs-long pulses at a repetition frequency of 76 MHz. The time correlation is provided by a PicoHarp 300 correlator (PicoQuant) triggered by a laser pulse. To avoid the pile-up effect, only 5% of the detected photon count was used to build the histograms.^17^

## 4 Results and Discussion

**Figure 1:**
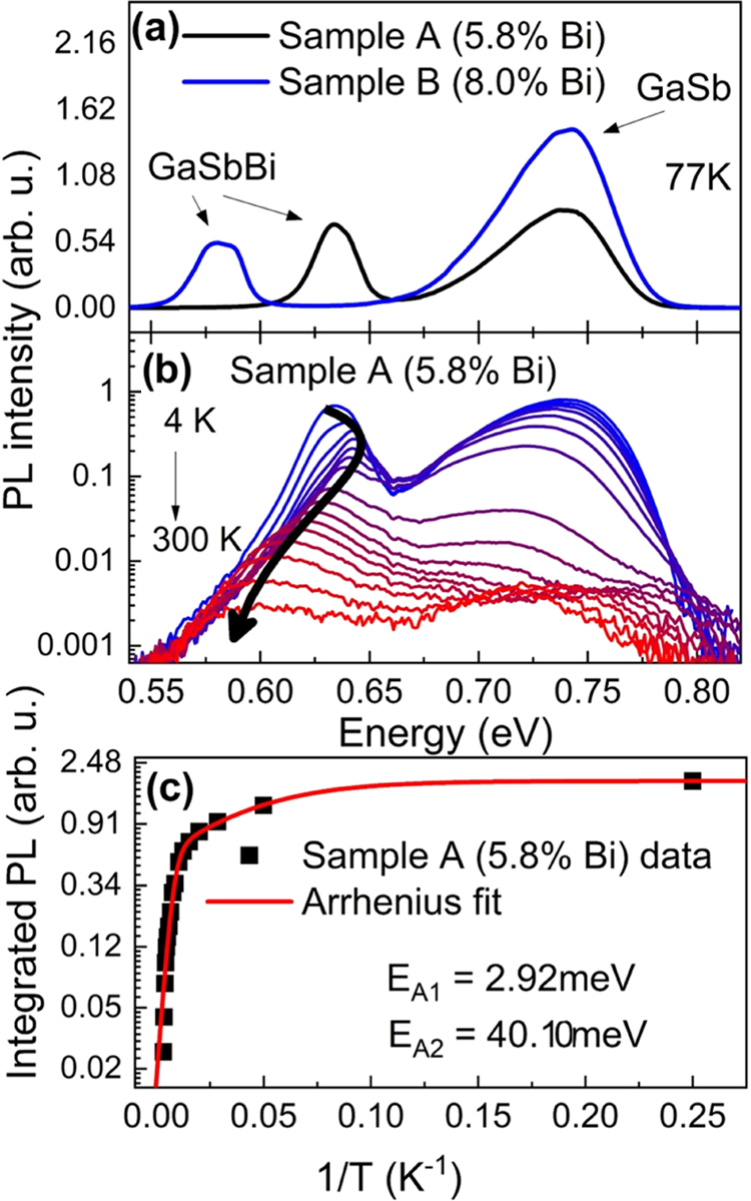
(a) Low-temperature PL spectra of samples A and B (5.8% Bi and 8.0% Bi). (b) Temperature-dependent PL spectra of sample A with linear temperature evolution, with a black arrow indicating the S-shaped behavior. (c) The Arrhenius plot of GaSbBi maxima with activation energies.

PL measurements for both samples showed two emission peaks (Figure 1a). First, a broad emission peak near 0.75 eV is observed in both samples and can be connected to the emission from the n-doped GaSb substrate. Second, at lower energies, both samples show additional peaks from the GaSbBi band edge around 0.63 and 0.58 eV for samples A and B, respectively. As the Bi content is increased from sample A to B, the band gap is reduced, and thus, the second peak is shifted to lower energy in sample B. Studies have shown that the bandgap shift can be estimated as ∼30 meV per % Bi, which is presented in Figure 1a. This confirms the values given in the literature.^18,19^ Temperature-resolved PL (see Figure 1b) recorded for sample A shows that the emission energy from the GaSbBi layer follows an S-shape (in the low-temperature range), which indicates the presence of localized states within the band gap of the GaSbBi matrix. These localized states can be connected to low-temperature growth defects and/or Bi atomic clustering or ordering. The same characteristics were also recorded for sample B (not shown). We further analyzed the data by fitting the Arrhenius equation to the emission intensity, which provides information about the activation energies of emission processes for the band edge (0.63 eV) optical signal, as shown in Figure 1c. We note that the GaSbBi PL signal is integrated from the low-energy side to a threshold energy of 0.65 eV for sample A and 0.62 eV for sample B, such that the contribution of the GaSb PL intensity is negligible and excluded from influencing the analysis. To obtain the maximum coverage of the fitted function to the data, it was necessary to use the double-Arrhenius function fit, given in eq 1, with the variables *I*_0_—initial intensity, *E*_a1,2_—activation energy, *B*_1,2_—pre-exponential factor, and *k*_B_—Boltzmann’s constant.^20^
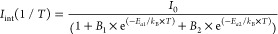
1

Activation energies from fitting the Arrhenius equation were as follows: 2.92 and 40.10 meV for sample A and 6.0 and 43.20 meV for sample B. The lower activation energies can be connected to the carriers escaping from the defect traps induced by Bi*_n_* clusters in the lattice structure.

**Figure 2:**
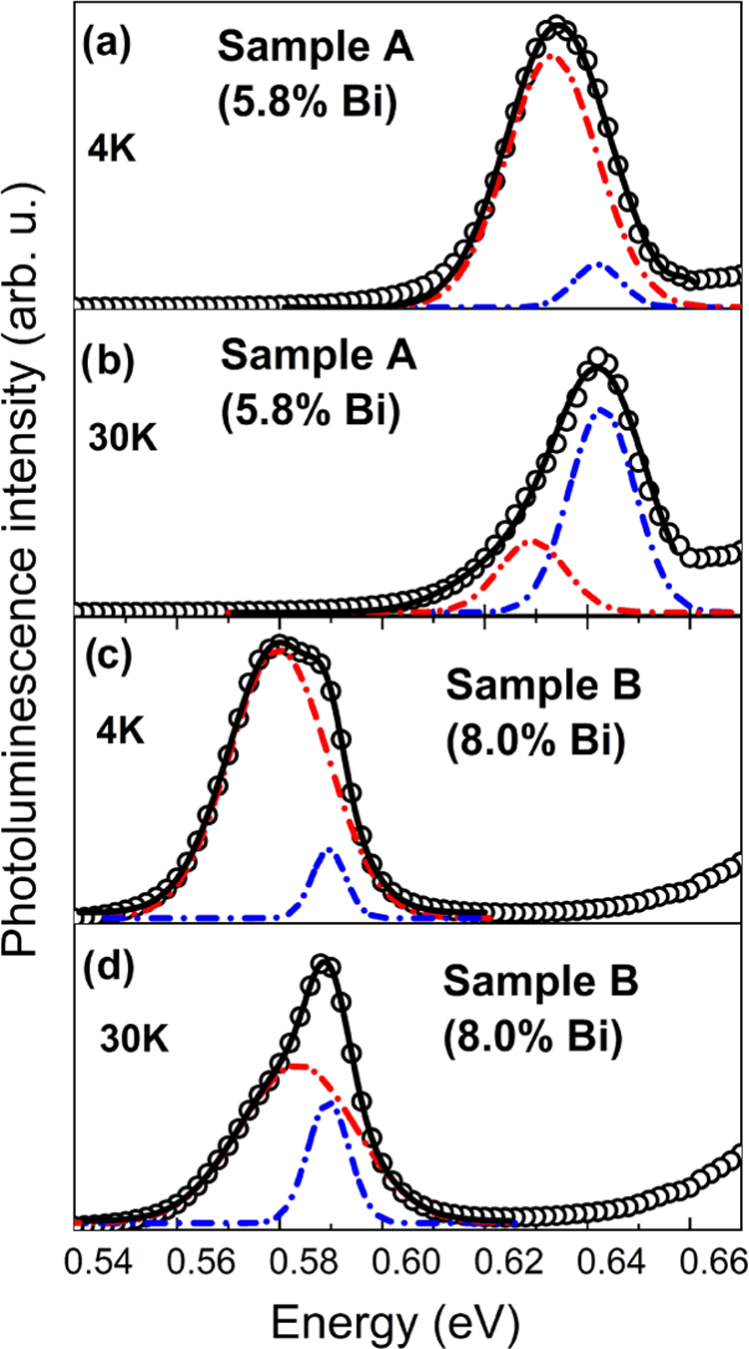
Temperature evolution of the measured PL spectra (black circles) and their components with a visible decrease in the defect-state emission (red) and increase in the GaSbBi band-edge emission (blue) for both 5.8% Bi (a, b) and 8.0% Bi (c, d) samples. The black curve indicates a summary of fit components.

As seen in Figure 2, the analyzed peak (related to the band edge of GaSbBi) consists of two maxima. The shape of the measured signal can be fitted using the double Gaussian function (red and blue dashed curves). A higher energy peak (blue) is connected to excitonic recombination, whereas a lower energy peak (red) might originate from the localized states connected to bismuth clustering. For sample A, by comparing panels a and b in Figure 2, we can see the changes in the contributions of both signals to the collective envelop of the optical feature. At 4 K, the process is mostly influenced by defect-related transitions. When the temperature increases, the total process becomes dominated by exciton recombination, while defect-related transitions are diminished. Analyzing the same situation for sample B (panels c and d), a similar evolution of the signal can be seen, but at 30 K, spectra remain dominated by the localized defect-related transition. This might be the first indication that an increase in the amount of bismuth atoms is strongly related to a higher number of localized centers or Bi*_n_* clustering.

The first conclusion from the analysis of both the Arrhenius fit and the low-temperature PL spectra suggests that the higher the bismuth atom content introduced in the crystalline lattice, the larger the amount of energy required to free the localized carriers from the defect traps. For sample A, the temperature equivalent of the defect activation energy (2.92 meV) is 34 K, which can be clearly observed in the temperature evolution of spectra. At 30 K, the emission from the state connected to the structural defects is much weaker. (Figure 2a,b) For sample B, the equivalent emission is stronger than that in the first sample at 30 K, and it is still observable up to 70 K. This also matches perfectly with the temperature calculated from the activation energy (6.0 meV). The higher activation energies (close to 40 meV) are significantly larger than the binding energy of excitons (few meV). This leads to the conclusion that, in our case, the PL signal is reduced by nonradiative processes.^21^

The analysis here is consistent with Raman spectroscopy measurements presented in a previous study on GaSbBi, where a vibrational mode was observed to be associated with Bi_2_ clustering in the lattice structure.^10^ This phenomenon could be significant for the optical properties of the samples presented in this article, causing the carrier trapping mechanism. We note that the investigated structures in this paper consist of thick layers of GaSbBi, with the PL being probed much deeper than the Raman signal. In fact, this effect could be diminished by the application of thin layers in low-dimensional systems such as quantum wells as the number of clusters probed by the PL would reduce.

**Figure 3:**
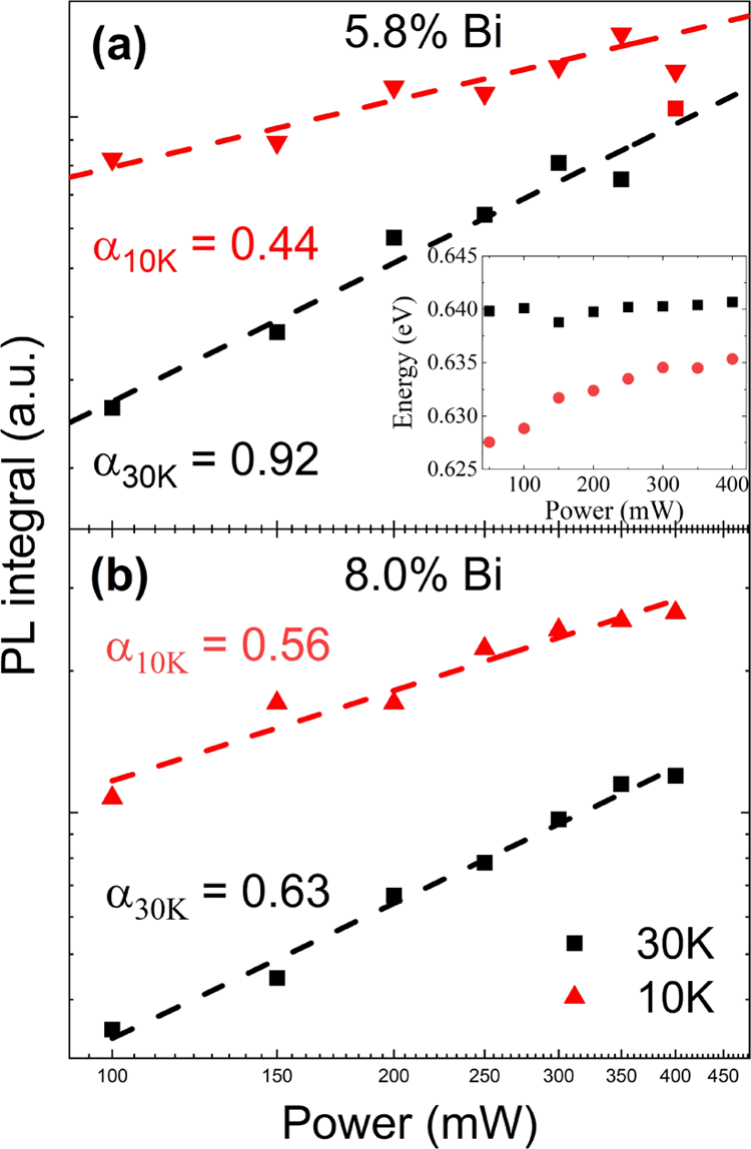
Power-dependent photoluminescence measured on two samples at two temperatures (10 and 30 K) presented in the log–log scale. After linear fitting, the α parameter for sample 5.8% Bi (a) at 10 K is below 1, but at 30 K, it is close to 1. In the 8.0% Bi sample (b), both parameters are below 1. The inset shows the blue-shift of PL.

For further verification of the previous analysis, an excitation-laser-power-dependent PL measurement was performed. The analysis of the spectra is shown in Figure 3a,b for samples A and B, respectively. Black squares and fitted curves denote the analysis performed for data obtained at 10 K, whereas red triangles and curves are related to the analysis of data obtained at 30 K. The performed fits are achieved according to the formula *I*_PL_(*P*) = β*P*_exc_^α^, where *I*_PL_ is the integrated PL intensity, *P* is the excitation power, and β and α are fitting parameters. β is the emission efficiency. The exponent α has the following values: α = 1 for excitonic recombination; α = 2 for free carrier recombination. The relation of 1 < α < 2 is true for the intermediate case where both free exciton and free carrier recombination take place,^22^ while α < 1 when emission is connected to impurity or defect-related emission.^23,24^ As can be seen, at 10 K, the α parameter is significantly lower than 1, which means that the emission at this temperature is dominated by the Bi clustering effect. At 30 K, the α parameter remains the same as that at 10 K for sample B, whereas for sample A, it increases to almost 1. The obtained results are consistent with conclusions obtained through the analysis of the temperature-dependent PL spectra, where we suspected that the bismuth clustering effect is stronger for sample B than for sample A. In addition, a blue-shift in the emission energy was observed, as seen in the inset of Figure 3a, which is consistent with the existence of localized states. At a temperature of 10 K, the blue-shift is more prominent, whereas at 30 K, it is significantly weaker.

**Figure 4:**
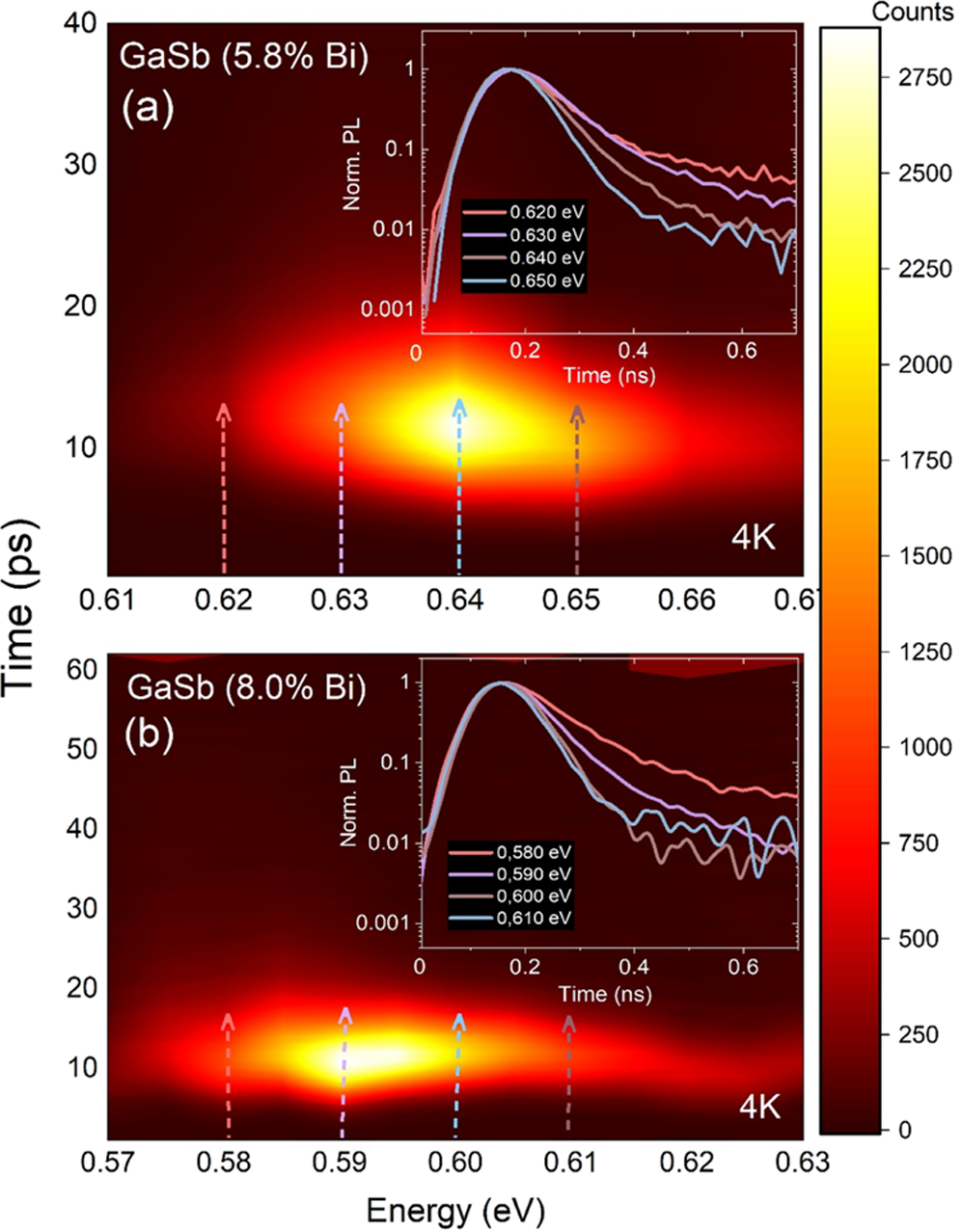
Time-resolved photoluminescence measurements for both samples at 4 K are presented as a map of the spectral and time regime. Panel (a) indicates TRPL for the sample with 5.8% Bi and panel (b) for 8.0% Bi. The insets present normalized PL decay with time for 4 different energies, shown with matching-colored arrows.

The main objective of this research was to determine the radiative PL lifetimes in this novel material system by utilizing time-resolved photoluminescence (TRPL). The mid-infrared regime made the measurements particularly problematic (e.g., due to a lack of streak cameras for this spectral range, which required the use of advanced techniques such as up-conversion^25^ or pump-probe measurements^26^). However, in our case, employing the SSPD detector made TRPL experiments achievable. The utilized setup provides information about the time evolution of the whole PL spectrum, which can be very convenient in analyzing the PL lifetime of different emission states. Figure 4 displays PL decay maps for sample A (panel a) and sample B (panel b). The counts represent the signal intensity change given by a color gradient; the *y*-axis represents the time scale for the decay process and the *x*-axis the energy scale of the PL signal. In the insets, the normalized PL signal decays are shown for four exemplary energies and indicated by vertical arrows on the main graphs. The decay time acquired from such spectra could be presented as . In our studies, when measurements are performed at 4 K, we assume that only radiative processes occur, which simplifies the equation, and by this way, we can determine the characteristic photoluminescence decay time constant. By fitting the time-resolved photoluminescence spectra using the formula , we can determine the PL lifetime constant τ_PL_.

**Figure 5:**
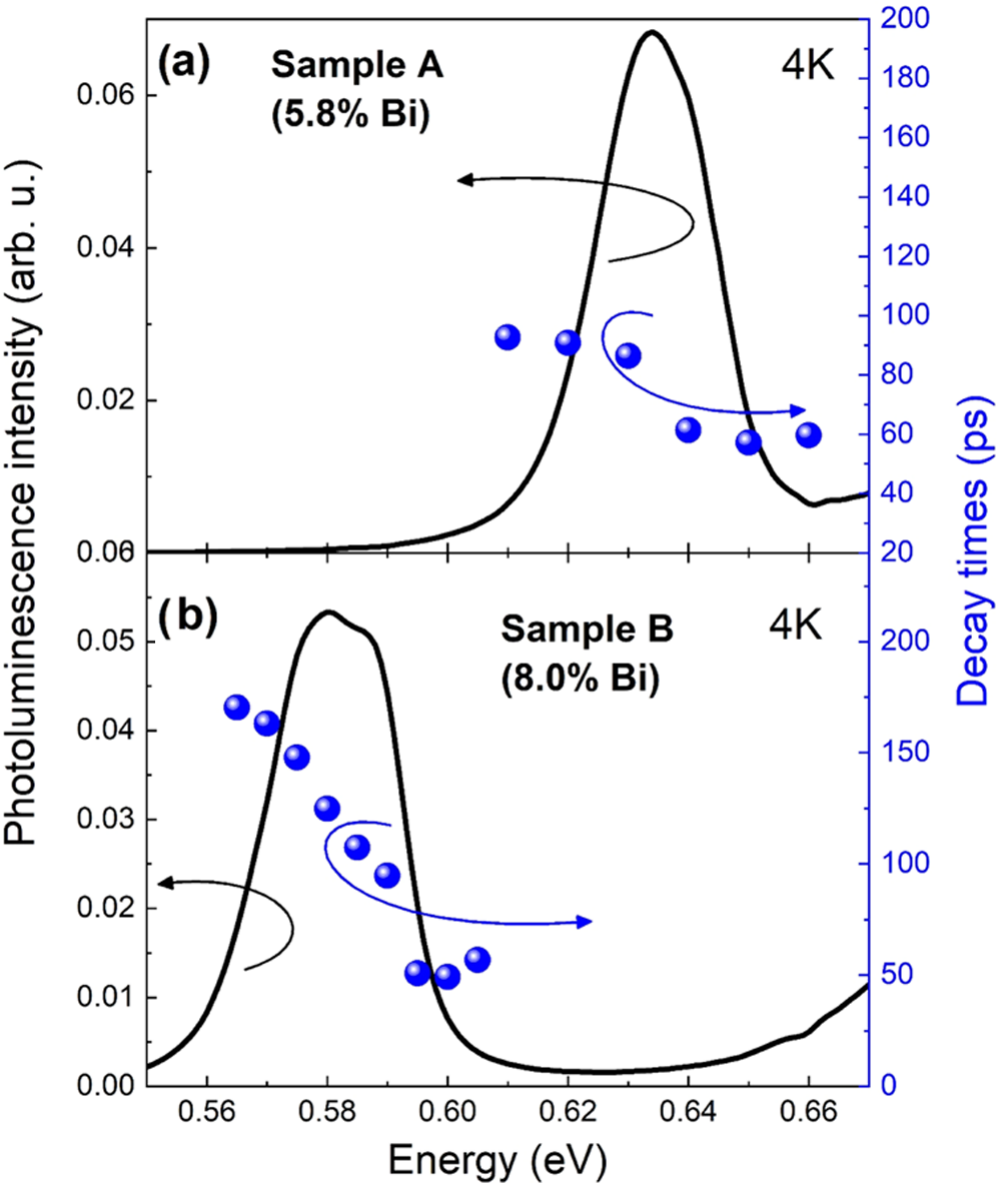
Photoluminescence spectrum (black) with indicated decay times (blue) of both samples. A significant visible increase in the lifetime near the energy corresponding to the defect-state emission. The obtained decay time also increases with the bismuth content (∼80 ps for 5.8% Bi and ∼150 ps for 8.0% Bi).

The determined PL lifetime in Figure 5 is presented as blue points, in addition to the PL spectra (black curve) overlaid for both samples. Starting the analysis from the higher energy (band edge) side of the signal, we see that the PL lifetime is similar for both samples, and its value varies from 50 to 60 ps. However, the lower energy part of the PL signal differs between the samples. The lifetime for sample A varies around 80–100 ps (Figure 5a), while that of sample B increases up to around 150–180 ps (Figure 5b). This phenomenon can be explained by the number of bismuth clusters in the lattice. The Bi concentration is linked to the Ga vacancy concentration,^27^ which drives the Bi cluster formation.^28^ Therefore, with increasing Bi concentration, there are more clusters that can trap carriers, which then increase the photoluminescence decay time. Similar observations were made regarding type-II GaAsSb/GaAs quantum wells,^29^ where the PL lifetime prolongation was connected to localized centers, resulting in the antimony segregation process at the GaAsSb/GaAs heterointerface.^30^ We also noted that for sample A, the time constant seems to be less dependent on the energy within the photoluminescence spectrum, which could be caused by the lower amount of bismuth clusters in measured structures. Nevertheless, this could also be explained by fewer measurement points acquired, as well as the use of similar scales on the graphs. In addition, the data acquired by measurement are influenced by some experimental errors, which became more significant in the case of less separated peaks (smaller amount of Bi clusters).

## 5 Conclusions

FTPL measurements were performed, confirming the energy gap of both samples, with 0.63 eV for the sample with 5.8% Bi and 0.58 eV for 8.0% Bi. Temperature-dependent photoluminescence allowed the determination of two activation energies for the emission peak, suggesting two recombination processes occurring in close proximity in terms of energy. These values were assigned to bismuth cluster trapping (2.92 and 6.04 meV) and to the recombination from the GaSbBi band edge (40.10 and 43.20 meV). Excitation-laser-power-dependent photoluminescence analysis showed that the power coefficient values at low temperatures were characteristic of defect occurrence, in contrast to spectra at temperatures above the activation energy of bismuth clustering. In addition, time-resolved photoluminescence measurements were performed as a function of energy dispersion, allowing the determination of the characteristic PL lifetime. Finally, the photoluminescence decay constant changed from 50 to 100 ps to 80–180 ps as the amount of incorporated bismuth atoms was increased.
